# Origami-inspired reprogrammable microactuator system

**DOI:** 10.1038/s41378-025-01026-x

**Published:** 2025-10-09

**Authors:** Vincent Gottwald, Lena Seigner, Makoto Ohtsuka, Rundong Jia, Pejman Shayanfard, Frank Wendler, Lars Bumke, Eckhard Quandt, Manfred Kohl

**Affiliations:** 1 Institute of Microstructure Technology, Karlsruhe Institute of Technology, Karlsruhe, Germany; 2 Institute of Multidisciplinary Research for Advanced Materials, Tohoku University, Sendai, Japan; 3 Institute of Materials Simulation, Friedrich-Alexander-Universität Erlangen-Nürnberg, Erlangen, Germany; 4 Department of Materials Science, Kiel University, Kiel, Germany

**Keywords:** Electrical and electronic engineering, Electronic properties and materials

## Abstract

A reprogrammable microactuator system is presented, consisting of antagonistic shape memory alloy (SMA) microactuators for bidirectional folding of miniature-scale tiles following the concept of origami. Additional integrated heatable soft-magnetic pads with low ferromagnetic transition temperature allow for control of magnetic latching forces. The strongly coupled thermo-mechanical and thermo-magnetic properties of the microactuator and magnetic subsystems are taken into account in a model-based design to enable their selective control by Joule heating. A procedure for local shape setting of the SMA microactuators is presented to adjust their memory shape at either maximum or minimum bending angle and, thus, to functionalize their performance as protagonists or antagonists. A microfabrication process is developed that takes the specific requirements for processing the various materials and structures into account. A demonstrator system consisting of four triangular tiles with an edge length of 500 µm and an angular range of about ±100° is introduced that is programmed to adopt the shape of a pyramid and later on reprogrammed to self-unlatch, self-unfold, and subsequently to adopt the shape of a table.

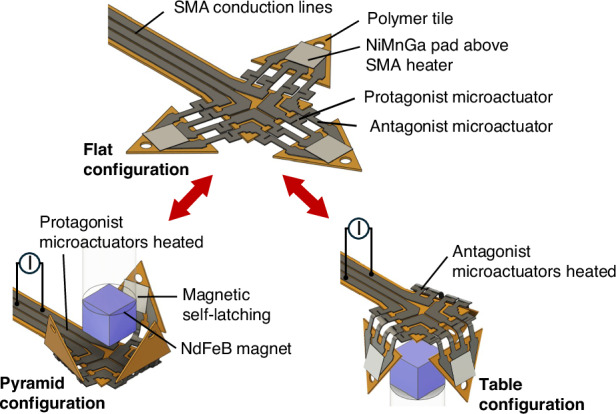

## Introduction

The principle of the traditional Japanese art of paper folding, Origami, is well known to enable the folding of a two-dimensional flat sheet consisting of rigid tiles and flexible hinges into a 3-dimensional (3D) shape. Transferring this concept to technical systems bears important potential, as complex 3D shapes can be realized after fabrication, allowing to adapt the system functionality to a given environment. Moreover, this leads to new mechanical, morphological, optical, or other properties that significantly enhance the system performance in various applications in science and engineering^1^. Various origami-inspired systems have been developed over many orders of magnitude, ranging from microrobotics^2^ and metamaterials^3,4^ to large deployable structures with lightweight design and high rigidity in avionics and space applications^5–7^. Particularly at the microscale, origami-inspired mechanisms offer unprecedented control over material properties and functionalities, enabling a wide range of potential applications from biomedical devices^8^ to advanced electronics^9^.

Self-folding on demand requires a suitable actuation concept. Various transducer materials such as hydrogels, shape memory polymers, liquid crystal elastomers, or magnetic soft materials have been investigated to fold rigid components^10^. At miniature scales, shape memory alloys (SMAs) are particularly suitable due to their extraordinary work density in the order of 10^7^ Jm^−3^, enabling large deformation and bending moments within restricted space that can be controlled by direct Joule heating^11–13^. Different SMA-based mechanisms have been developed, comprising linear^14,15^, torsional^16^, and folding principles^17,18^.

Most origami-inspired mechanisms allow for unidirectional self-folding to a programmed 3D shape, which are often referred to as programmable matter or robogami^19^. The combined interaction of the folded components gives rise to enhanced or novel mechanical properties, including multistability^20,21^, semi-soft behavior, and high adaptivity^22^. In particular, introducing multistability avoids ambiguous folding branches and stabilizes the final 3D shape. Possible latching mechanisms are predeflected membranes or beams showing snapping instability^23^ or magnetic latching^24^.

However, in many cases, self-folding origami-inspired structures are unidirectional and, thus, they show limited reconfiguration once the 3D structure is fixed after actuation, and manual intervention becomes a necessity^25–27^. Therefore, an important extension of programmable matter is the potential to reconfigure the 3D shape reversibly by self-unlatching and self-unfolding, which leads to the concept of reprogrammable matter. In order to self-unlatch the 3D structure, a release mechanism is required, while self-unfolding requires additional actuators for resetting before a new folding sequence and subsequent latching of a new reprogrammed shape can be accomplished. To date, only a limited number of reprogrammable origami-inspired systems have been fabricated and investigated^28^. Current reconfigurable SMA-based systems with multiple tiles are restricted to macro-scale sizes^29^ due to limits in fabrication technology and thermal design. However, the concept of reprogrammability becomes a necessity upon miniaturization to millimeter dimensions and below, as manual reconfiguration is hardly possible.

In this work, we design, fabricate, and characterize a reprogrammable origami-inspired microactuator system. Major components are antagonistic SMA microactuators for bidirectional folding of miniature-scale tiles with 500 µm lateral dimensions and heatable soft-magnetic pads for reversible magnetic latching. The challenges include the microactuator design and simulation taking into account thermal, mechanical, and magnetic coupling effects, the microfabrication of the multi-material system, and the local heat treatment of the SMA microactuators under constraint conditions to set their memory shape individually.

## Results

### Layout and operation principle

**Fig. 1:**
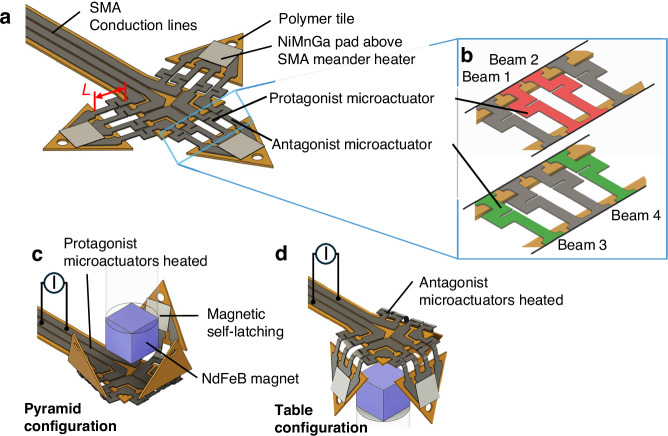
**Layout of the reprogrammable origami-inspired microactuator system consisting of a centrally located polymer tile and three movable polymer tiles**. The tiles are interconnected with protagonist and antagonist SMA microactuators that can be controlled via SMA conduction lines forming two separate electrical circuits. The antagonist microactuators are interconnected in addition with SMA meander structures on each tile for indirect heating of soft-magnetic pads located above. This enables controlling the magnetic latching forces between the pads and an external miniature magnet. **a** Initial flat configuration, **b** close-up of protagonist and antagonist microactuators with double-beam layout, **c** pyramid configuration obtained by Joule heating the protagonist microactuators, **d** table configuration obtained by Joule heating the antagonist microactuators

The intended functionalities of the microactuator system include the bidirectional folding of several tiles with lateral dimensions of 500 µm as well as their latching and unlatching on demand to adopt, maintain, and reset different 3D shapes that can thus be reprogrammed. Figure 1 shows the layout of the microactuator system. Major components are three pairs of antagonistic SMA microactuators that allow for bidirectional bending in the out-of-plane direction, either upwards or downwards, by selectively addressing the protagonist or antagonist microactuators. Each pair of antagonistic SMA microactuators connects two rigid polymer tiles with a triangular shape. The centrally located tile serves as a basis to interconnect and power the outer tiles. The SMA microactuators consist of a TiNiCu double beam that can be Joule heated directly with an electrical current to transform into its memorized shape, which has to be adjusted initially by thermo-mechanical treatment. Wing-like structures in the double beam improve temperature homogeneity and compliance to increase the angular range of bending^30^. The memorized shapes of the antagonistic SMA microactuators need to be adjusted in opposite bending directions in order to fold up- or downwards, depending on which microactuator is Joule heated. The TiNiCu beams of the protagonist and antagonist microactuators are interconnected by TiNiCu conduction lines forming separate electrical circuits. The TiNiCu beams forming the antagonist microactuators are interconnected in addition to TiNiCu meander structures on each tile. They form local heaters for indirect heating of soft-magnetic NiMnGa pads located above to induce a ferromagnetic transition. Thus, local heating of the NiMnGa pads allows to switch of the magnetic attraction force between the NiMnGa pads and the NdFeB permanent magnets located centrally above and below the microactuator system.

By switching on the electrical current in the conduction lines of the protagonist microactuators, they transform into their memorized shape and bend upwards. Once the NiMnGa pads get close to the permanent magnet, latching sets in and maintains the folded state. In the present layout, all three protagonists are bent upwards at the same time, as sketched in Fig. 1b to form a pyramid. This configuration is maintained after the heating current is switched off. However, it can be reset on demand by selectively heating the antagonist microactuators, which generate a shape recovery force directed away from the permanent magnet. At the same time, the local heaters are activated, causing a ferromagnetic transition of the NiMnGa pads and, thus, the magnetic attraction force is switched off. Consequently, the tiles self-unlatch and the antagonist microactuators bend downwards at the same time. Thereby, the tiles self-unfold and eventually self-fold in the opposite out-of-plane direction, forming a table as sketched in Fig. 1c. By tuning the heating current and time, self-latching is used to maintain the table configuration. The microactuator system undergoes this sequence on demand by selectively heating the protagonist or the antagonist microactuators. Thus, the 3D shape of the system can be reprogrammed without any manual intervention.

### Materials properties

**Fig. 2:**
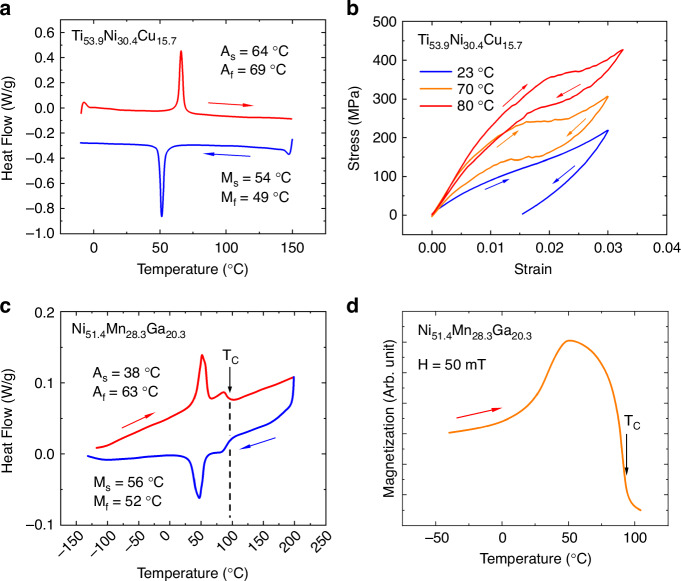
**Material properties of the Ti**_**53.9**_**Ni**_**30.4**_**Cu**_**15.7**_ **and Ni**_**51.4**_**Mn**_**28.3**_**Ga**_**20.3**_ **films used in this investigation**. **a** Differential scanning calorimetry (DSC) measurement of a TiNiCu film of 10 µm thickness after rapid thermal annealing at 700 °C for 15 min; **b** stress–strain characteristics of a TiNiCu tensile test specimen of 10 µm thickness at different temperatures in the range from 23 to 80 °C determined at a strain rate of 10^−4^ s^−1^; **c** DSC measurement of a NiMnGa film of 5 µm thickness after thermal annealing at 800 °C for 10 h; **d** magnetization versus temperature characteristic of a NiMnGa film of 5 µm thickness at a magnetic flux density of 50 mT. Legend: *M*_*s/f*_ - start and finish temperatures of martensite phase transformation, *A*_*s/f*_ - start and finish temperatures of reverse transformation, *T*_*C*_ - Curie temperature of 94 °C

The SMA materials used for microactuation are magnetron-sputtered TiNiCu films of 5 and 10 µm thickness. After substrate release, the films are heat-treated by rapid thermal annealing at 700 °C for 15 min in a vacuum chamber to crystallize the films and to adjust the martensitic phase transformation^31,32^. The TiNiCu films are of special interest for long lifetime applications due to their small grain size in the sub-µm range in combination with coherent precipitates and a good crystallographic compatibility over a wide compositional variation. A detailed investigation of the different factors on functional and structural fatigue in TiNiCu can be found in ref. ^32^. The phase transformation temperatures of the films are investigated by differential scanning calorimetry (DSC) at a constant heating and cooling rate of 10 Kmin^−1^. Figure 2a shows the DSC measurement of a TiNiCu film of 10 µm thickness after heat treatment. The start/finish temperatures of the austenite and martensite phase transformation, *A*_*s/f*_ and *M*_*s/f*_, are determined by the tangential method to be 64/69 °C and 54/49 °C, respectively. In this work, the one-way effect of TiNiCu is utilized for actuation. In the martensitic state at a temperature below *M*_*f*_, the TiNiCu film can be easily deformed by mechanical load. Upon heating the TiNiCu film to a temperature exceeding *A*_*f*_, the residual strain is reset, enabling the TiNiCu film to regain its memory shape.

A tensile test setup is used to investigate the stress–strain characteristics of TiNiCu test specimens having a dogbone shape with a length *l*, width *w*, and thickness *t* of 5.5 mm, 500 µm, and 10 µm, respectively. The loading rate is set to 10^−4^ s^−1^ to maintain quasi-stationary conditions during loading and unloading. Figure 2b shows the stress–strain characteristics of a TiNiCu test specimen in the strain range up to 3.25 % at different temperatures starting from room temperature up to 80 °C. Given that the SMA film is in martensitic state at room temperature, the stress–strain characteristics exhibit a typical nonlinear quasi-plastic behavior, caused by the accommodation of martensite variants upon loading. After reaching the strain limit and subsequent load release, a remanent strain occurs, which can be reset by heating the specimen above the austenite finish temperature *A*_*f*_.

At 70 °C, the film exhibits superelasticity, which is characterized by an initial linear stress increase followed by a stress plateau due to stress-induced martensitic phase transformation. Upon unloading, the strain is reset to its initial state. At 80 °C, the stress plateau sets in at 2 % strain at a stress of about 360 MPa. Thus, the maximum shape recovery stress at 2 % strain, given by the stress change upon heating from room temperature to 80 °C, is about 230 MPa.

The materials used for reversible magnetic latching are Heusler alloy NiMnGa films of 5 µm thickness. The films are deposited onto a polyvinyl alcohol substrate using magnetron sputtering at 200 W. After substrate release, the films are heat-treated under vacuum conditions at 800 °C for 10 h. Details on the fabrication and the resulting properties of the films can be found in refs. ^33–35^. Figure 2c shows a DSC measurement of a NiMnGa film after heat treatment at a constant heating and cooling rate to evaluate the characteristic temperatures of the austenite and martensite phase transformation. The start/finish temperatures *A*_*s/f*_ and *M*_*s/f*_ are determined by the tangential method to be 38/63 °C and 56/52 °C, respectively. The jump-like feature is due to the second-order ferromagnetic transition at the Curie temperature *T*_*C*_ which is determined to be 94 °C. A vibrating sample magnetometer is used to perform temperature-dependent magnetization measurements. Figure 2d shows a magnetization versus temperature characteristic of a NiMnGa film upon heating at a low magnetic field of 50 mT. Between 70 and 100 °C, the magnetization exhibits a large drop due to the ferromagnetic transition. The value of *T*_*C*_ is determined by the tangential method.

### Design and simulation

**Fig. 3:**
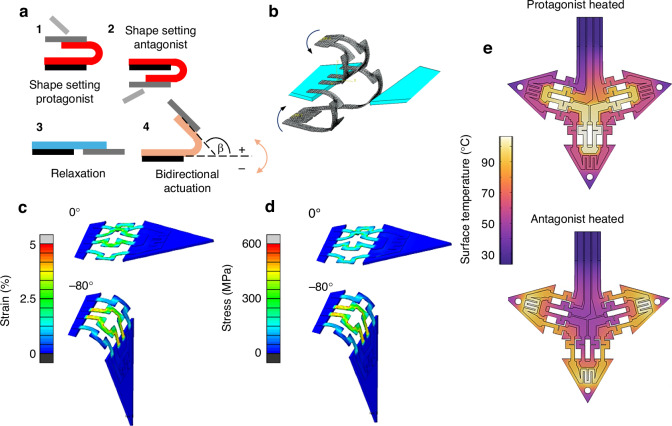
**Coupled finite element simulations of the origami-inspired reprogrammable microactuator system**. **a**, **b** The procedure for local shape setting of the SMA microactuators in the experiment and simulation, respectively. The black arrows denote the imposed out-of-plane rotational boundary condition. **c**, **d** The evolution of strain and stress profile is shown upon selective Joule heating of the antagonist microactuator, respectively, starting from flat state after preprocessing (top) and reaching -80° in heated state (bottom). **e** Simulated temperature profiles caused by selective Joule heating of the protagonist (top) and the antagonist microactuator (bottom) under quasi-stationary conditions at a heating power of 35 mW

Due to the close vicinity of conduction lines, microactuators, local heaters, and soft-magnetic pads, the electro-thermal, mechanical, and magnetic properties are strongly coupled and, thus, have to be designed in parallel. The electro-thermal and mechanical properties of the SMA microactuators are determined by local heat treatment of the SMA microactuators under constraint conditions required to set their memory shape individually. Here, local shape setting is investigated by applying direct Joule heating while fixing the microactuators in a bent state, which can be performed on a sub-millimeter scale using micromanipulators. As illustrated in Fig. 3a, the microactuators are first bent and fixed at a pre-defined angle of 180° at a pre-defined bending radius. Subsequently, selective Joule heating is performed at different time intervals for different values of electrical power. The obtained performance of electrical resistance and maximum bending angles is summarized as a function of heat treatment time at different heat treatment temperatures in Figs. S1 and S2 in the Supplementary Material, respectively. Based on this investigation, we select the final heat treatment temperature and time to 485 °C and 60 s, respectively. In this case, surface oxidation is limited, and thermal cross-coupling between neighboring protagonist and antagonist microactuators is avoided.

In addition, the maximum strain in the fully bent state should be kept below a critical limit to avoid plastic strain and lifetime issues^36^. The material-dependent strain limit, in turn, poses a lower limit on the bending radius and length of the microactuators. A rough estimate on the lower limit of length *L*_*min*_ can be made by assuming ideal bending conditions with a neutral axis centered in the beam cross-section. In this case, the length *L*_*min*_ can be approximated to be:

1 $${L}_{min}=\frac{{t}*{\beta}}{{2}*{\varepsilon}_{max}},$$

whereby the thickness of the SMA microactuator, the folding angle, and the maximum strain are labeled by *t, ß*, and *ε*_*max*_, respectively. For an optimal use of the shape memory effect, the strain should also be larger than the minimal strain to induce detwinning^37^. To detwin a maximum volume fraction of martensite in the folded beams, the optimal length should be close to *L*_*min*_. For a folding angle *ß* of 90°, a maximum strain of 2 %, and SMA film thicknesses of 5 and 10 µm, this mechanical design criterion leads to minimal lengths *L*_*min*_ of 196 µm and 392 µm as well as minimal bending radii of 62.5 µm and 125 µm, respectively. The local heaters are designed for an area of 250 × 250 µm^2^ to generate a maximum temperature of about 110 °C, required to surpass the ferromagnetic transition temperature of NiMnGa. Due to the small thickness of the NiMnGa pads of 5 µm, the ferromagnetic transition is induced via rapid heat transfer. At the same time, the temperature of the opposing SMA microactuator should remain below the austenite start temperature *A*_*s*_ of 77 °C to avoid bending actuation.

A thermodynamic model has been developed to simulate the coupled performance of the protagonist and antagonist microactuators, which is presented in the section on Materials and Methods. Table S1 in the Supplementary Material summarizes the thermal and electrical material parameters used in these simulations. Different from the experimental procedure, the shape setting of the SMA microactuators in the simulations involves a preprocessing step, which is illustrated in Fig. 3b. First, the flat microactuators are connected only to the central tile and bent towards +180° (protagonist) and −180° (antagonist) by imposing an out-of-plane rotational boundary condition to a reference point tied on the top-most surface area of the SMA microactuators, see arrows in Fig. 3b. Second, these close-folded geometries are used as the memorized geometry and imported to the next simulation step, where they are folded reversely and subsequently locked to the movable tile in flat condition by a surface-to-surface contact. Thereby, martensite reorients, and stress builds up. The resulting state represents the functionalized bidirectional microactuator, which is characterized by a metastable equilibrium between the antagonist and protagonist (Fig. 3c and d, top). Figures 3c and d also show the effect of selective Joule heating of the antagonist on the evolution of strain and stress profiles, respectively. While the antagonist is recovering its memorized shape, the bending angle and concomitantly the strain and stress are decreasing. Ultimately, the bending angle reaches about −80°, which is determined by the force equilibrium of the heated antagonist and unheated protagonist microactuator.

The effects of thermal cross-coupling upon selective Joule heating are investigated using a heat-transfer model implemented in COMSOL Multiphysics. Thereby, the thermal conductivity is approximated by the Wiedemann-Franz law, describing the heat conductivity as a function of electrical conductivity^38^. Figure 3e shows simulated surface temperature profiles caused by selective Joule heating of the protagonist or the antagonist microactuator under quasi-stationary conditions for a width of the double beams of 60 µm and a gap in between the beams of 100 µm. When the protagonist microactuator is heated with a current of 25 mA, the temperature difference with respect to the antagonist microactuator is between 40 and 50 °C, while all beams of the protagonist microactuator are heated above the *A*_*f*_ temperature. In contrast, the temperature difference between these beams differs by less than 15 K. Similar temperature differences are obtained when the antagonist microactuator is heated with a current of 25 mA. In this case, the current also flows through the local heater, which reaches a maximum temperature of 110 °C (Fig. 3e, lower image), which is above the Curie temperature of the magnetic film material. Further information on the influence of the thicknesses of polymer tile and SMA film, as well as the effect of scaling the overall size of the microactuator system on the required electrical heating power and thermal cross-coupling, is presented in Figs. S3 and S4 in the Supplementary Material.

### Microfabrication

**Fig. 4:**
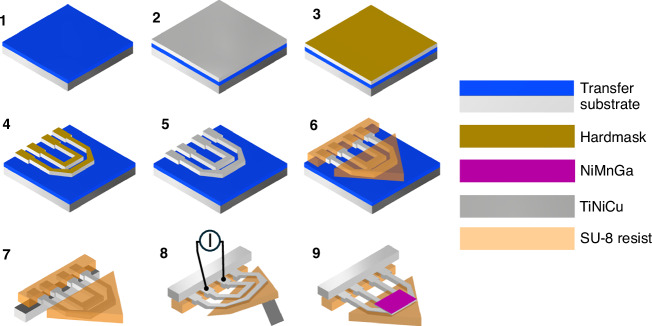
**Process flow for microfabrication of the origami-inspired reprogrammable microactuator system**. (1) Preparation of a transfer substrate using a heat-release polymer film laminated on a Si substrate, (2) bonding of the TiNiCu film onto the transfer substrate, (3) evaporation of a Cr and Au layer, (4) first UV lithography of Au/Cr hard mask and wet-etching of TiNiCu film, (5) removal of hard mask, (6) second UV lithography of SU-8 tiles, (7) release of the two-layer system, (8) shape setting, (9) third UV lithography and integration of NiMnGa pads

A scalable process has been developed for the fabrication of the microactuator system. As illustrated in Fig. 4, the magnetron-sputtered and annealed TiNiCu films are bonded onto a transfer substrate consisting of a heat-release polymer foil and a silicon substrate (2). An Au/Cr layer is evaporated (Leybold UNIVEX 400) onto the TiNiCu film (3), which is micromachined in a first UV lithography step to prepare the Au/Cr hard mask for the subsequent wet-etching step (4). The UV lithography step includes the spin-coating (Primus STT15) of a positive resist layer of 1 µm thickness, subsequent pre-exposure baking at 95 °C for 3 min, and direct laser exposure (Heidelberg Instruments DLW66fs) at a power of 45 mW and a dose of 80 mJ at 405 nm wavelength. The wet-etching step includes the subsequent removal of the unprotected Au and Cr areas, followed by the removal of the unprotected TiNiCu areas using a HF:HNO_3_:DI solution. Subsequently, the hard mask is removed by wet-etching (5). The rigid tiles are made of SU-8 negative photoresist, which is widely used in microtechnology, offering high temperature stability and low thermal conductivity^39^. The tiles are micromachined in a second optical lithography step (6). Thereby, SU-8 photoresist is spin-coated on top of the TiNiCu microstructure with a thickness of 20 µm, exposed using an aligned polymer photomask, and developed. The resulting two-layer system is released from the transfer substrate at an elevated temperature (7). At this point, the shape setting procedure is applied as introduced before. Finally, soft-magnetic NiMnGa pads are prepared from magnetron-sputtered and annealed NiMnGa films by wet-etching and integrated onto the TiNiCu meander structures (local heaters) by a pick-and-place procedure. For latching experiments, an external NdFeB magnet of 400 × 400 × 400 µm^3^ size is used, having a flux density of 1.24 T.

### Performance of the SMA microactuator system

**Fig. 5:**
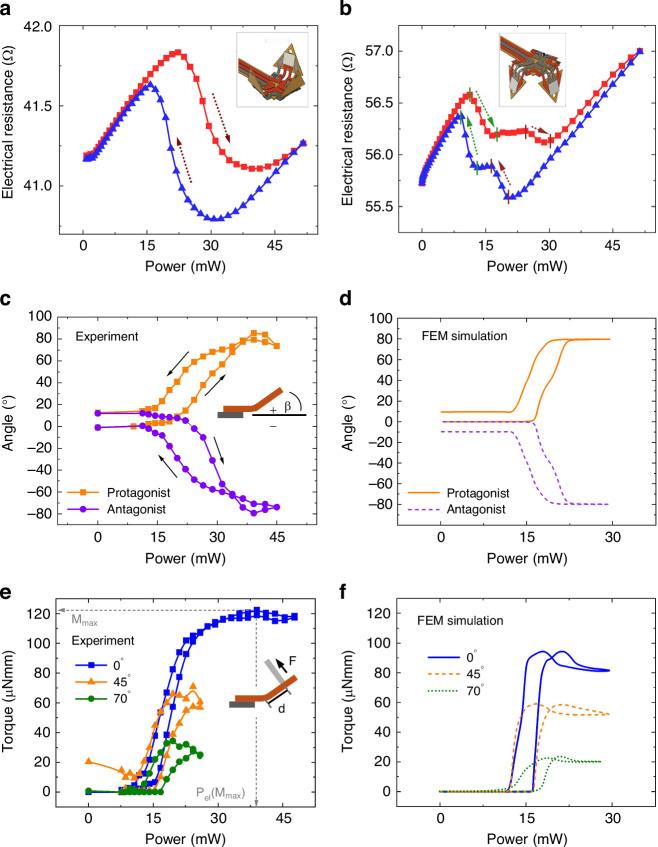
**Performance of the SMA microactuator system as a function of heating power**. Electrical resistance of the protagonist microactuators (**a**) showing a phase transformation in a single step, and of the antagonist microactuators and local heaters (**b**) showing two separate steps of phase transformation. The insets indicate the corresponding folding state of the tiles, whereby the red color indicates the Joule-heated electrical circuit. **c** Experimentally determined folding angle versus power characteristics showing maximum and minimum values of about ±80° and **d** corresponding simulated characteristics determined by coupled FEM simulations. **e** Experimentally determined torque versus power characteristics of a protagonist microactuator at three different angles, as indicated, and **f** corresponding simulated characteristics determined by coupled FEM simulations

Electrical resistance measurements are performed by the four-point measurement method to characterize the phase transformation of the protagonist and antagonist SMA microactuators as a function of heating power. The microactuators are Joule heated by ramping the electrical current stepwise up- and downwards under quasi-stationary conditions, allowing for sufficient waiting periods between each step. Before the experiment, the microactuators are cycled several times to achieve a reproducible performance. Figure 5a shows the electrical resistance versus power characteristics of the protagonist microactuators, revealing the typical hysteresis loop due to the martensitic phase transformation. In contrast, the electrical resistance shown in Fig. 5b comprises the contributions of the antagonist microactuators and the meander structures of the local heaters. Therefore, the overall resistance at zero power is 55.4 Ω, being significantly larger than that of the protagonist microactuators. For increasing electrical power, the electrical resistance drops in two stages that are well separated by a plateau in between. The first stage occurring at about 13 mW is due to the phase transformation of the local heater, which has a large resistance and, thus, requires a low power to reach the *A*_*f*_ temperature upon Joule heating. The second stage, at about 22 mW, is caused by the phase transformation of the antagonist microactuators. In addition, the electrical resistances of the unheated electrical circuits are monitored simultaneously to investigate thermal cross-coupling. These measurements reveal critical power levels, above which phase transformations occur due to heat transfer between the microactuators, as can be seen in Fig. S4 in the Supplementary Material. When Joule heating the antagonist microactuators, the critical power level is reached at 32 mW.

The angular range of bidirectional folding and the corresponding torque of bending actuation are important metrics for potential applications of the reprogrammable microactuator system. Figure 5c shows experimental results of folding angle versus power of the protagonist and antagonist microactuators. The folding angles are determined by digital image processing using a microscope camera. For increasing power, the folding angles increase/decrease above critical power values required to induce phase transformation and reach maximum/minimum values of about ±80°. When decreasing the power, a hysteresis occurs, reflecting the kinetics of reverse transformation in the active SMA microactuators and the torque of the opposing SMA microactuators in the unheated state. The maximum angle resets almost completely with a remaining offset in the range of 0°–10°. Thereby, the course of the folding angles of the protagonist and antagonist microactuators is nearly symmetrical. The simulation results shown in Fig. 5d reveal a similar course of the folding angles. In addition, the maximum and minimum angles, as well as the hysteresis, are in good agreement with the experimental results.

The torque of the SMA microactuators is determined experimentally through the blocking force that is generated during shape recovery. For this purpose, the SMA microactuators are bent to adjust different folding angles below a load cell with one end fixed onto a substrate as sketched in the inset. The shape recovery during heating is blocked by the load cell, and the reaction force is measured. The corresponding torque is calculated by multiplying the measured blocking force of the load cell by the distance *d* between the contact point of the load cell and the double-beam center. Figure 5e shows the experimentally determined torque versus power characteristics of a single protagonist microactuator at three different folding angles. Again, a hysteresis is observed, reflecting the difference between the forward and reverse phase transformation. The torque increases for increasing power until a maximum is reached. The maximum torque is about 122 µNmm at a folding angle of 0°. Lower values of maximum torque are observed when increasing the folding angle. At 45°, the torque reaches a maximum of 72 µNmm at a power of 25 mW, while at 70°, it reduces further to a maximum of 35 µNmm at 16 mW. At large heating power above 40 mW, the torque decreases again due to the onset of thermal cross-coupling, which causes counter-actuation by the antagonist microactuator. The corresponding simulation results are shown in Fig. 5f. Direct comparison of Fig. 5c–f reveals that the simulated heating power is lower than the experimental results. This deviation is attributed to contact resistances, which are not taken into account in the simulations, as well as deviations in dimensions. The simulation result also reveals a maximal torque of about 90 µNmm, which is 30 µNmm lower compared to the experimental result. This deviation is attributed to different load points. In the simulation, this point is taken directly at the beam end. In the experiment, a measuring pin with a diameter of 250 µm is placed in the center of the tile.

### Performance of the magnetic latching system

**Fig. 6:**
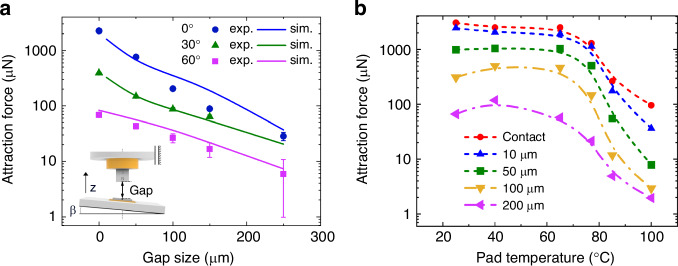
Magnetic attraction force between a miniature NdFeB hard magnet and a soft-magnetic NiMnGa pad integrated on a tile. The size of the magnet is 400 × 400 × 400 µm^3^. **a** Force versus gap size in z-direction at different angular orientations *β* as sketched for a pad size of 350 × 350 µm^2^ ; **b** force versus temperature at various gap sizes as indicated for a pad size of 1 × 1 mm^2^  at an angle of 0°

Magnetic latching is investigated by positioning a miniature NdFeB hard magnet of 400 × 400 × 400 µm^3^ size opposite to a tile with an integrated heatable soft-magnetic NiMnGa pad having a lateral size of 350 × 350 µm² and a thickness of 5 µm. The magnetic attraction force is investigated by varying the distance and angle between the magnet and tile as illustrated in the inset of Fig. 6a. The magnet is attached to a linear bearing so that the load cell can take up only the force component in the axial (z-)direction. The force shows a notable decline for increasing gap size and angle. The force maximum at contact is about 2.2 mN for the pad size of 350 × 350 µm². When increasing the pad size up to 1 × 1 mm², the contact force increases up to about 3 mN, as shown in Fig. 6b. Our studies on a NiMnGa pad size of 1 × 1 mm² reveal the same trend of distance and angle dependencies as shown in Fig. 6a.

Magnetostatic simulations in COMSOL Multiphysics are conducted to determine a position-parametrized form of the magnetic attraction force, which is used in the finite element model of the microactuator system. A multi-output regression procedure is set up, which is described in the Supplementary Material. Figure 6a shows a comparison between experimental data points and simulated latching forces, which rely on magnetization data at various external fields, recorded for different NiMnGa films^33^. Fig. S6 in the Supplementary Material further compares the simulated magnetic latching forces and model predictions at different polynomial degrees. Low-degree polynomials fail to capture the complexity of the data, while high-degree polynomials tend to overfit, resulting in unrealistic fluctuations. Overall, the used polynomial expansion provides a good fit to the experimental data.

Figure 6b shows the temperature dependence of magnetic attraction force for a pad size of 1 × 1 mm^2^ at various distances between the magnet and tile. In this case, the tile is placed on a temperature-controlled stage at an angle of 0°, while the attraction force is determined in the z-direction. The magnetic attraction forces start to decline above a critical temperature of about 70 °C and strongly decrease for all distances due to the ferromagnetic transition. The low ferromagnetic transition temperature allows for local heating at low heating power to switch the magnetic attraction force on demand. The attraction forces are restored by cooling the pads below 60 °C.

### Demonstrator system

**Fig. 7:**
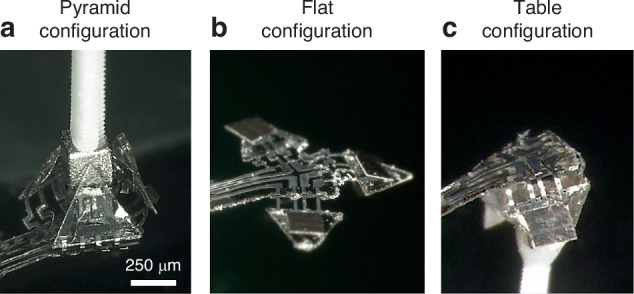
**Demonstrator of a reprogrammable origami-inspired microactuator system**. The edge length of the tiles is 500 µm, the lateral dimensions of the NiMnGa pads are 350 × 350 µm². **a** Pyramid configuration after self-folding and magnetic latching of the upfolded tiles; **b** intermediate flat configuration after self-unlatching and self-unfolding; **c** table configuration after subsequent self-folding and magnetic latching of the downfolded tiles

The final demonstrator system is realized by combining the SMA microactuator and magnetic latching subsystems. Figure 7a shows the system in the pyramid configuration, in which all three tiles are self-folded upwards by Joule heating the protagonist microactuators and subsequent magnetic latching of the upfolded tiles. The permanent magnet in this demonstrator is bonded to a 3D-printed polymer pin, which can be positioned in the z-direction perpendicular to the base tile to adjust the gap between the magnet and the three NiMnGa pads. Once the maximum bending angle of about +80° is reached, the gap is reduced until the latching force begins to dominate at a critical gap size of 160 µm, and the tiles snap towards their end position. This configuration is maintained after the heating power of the protagonist microactuators is switched off.

**Fig. 8:**
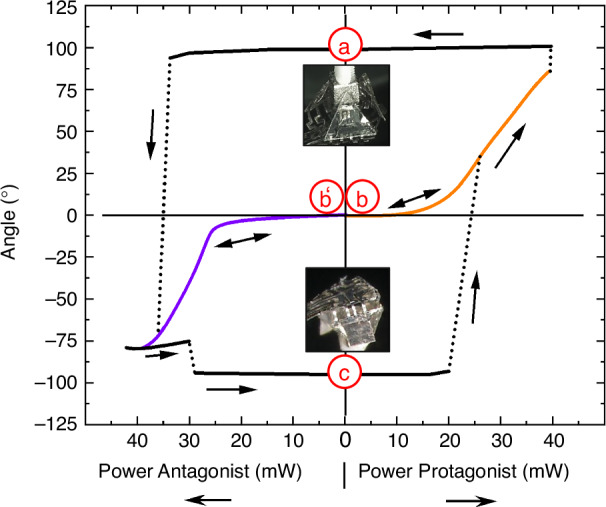
**Angle versus power characteristics of the reprogrammable origami-inspired microactuator system**. The power of the protagonist and antagonist is depicted in the right and left direction, respectively. **a** Pyramid configuration; **b**, **b’** intermediate flat configurations obtained after self-unfolding, whereby the power is switched off before the critical bending angles for magnetic latching of +80° and -80° are reached, respectively. The corresponding characteristics are highlighted in color; **c** table configuration. The dashed lines indicate abrupt angular changes

Figure 7c shows the system in the table configuration, in which all tiles are bent downwards in the lower magnetically latched position. In order to reconfigure the 3D shape from a pyramid to a table, the system first has to self-unlatch and self-unfold before self-folding to the table configuration is accomplished. The course of shape reconfiguration is shown in Fig. 8.

Starting from the pyramid configuration (a), reconfiguration is achieved by locally heating the NiMnGa pads above the ferromagnetic transition to strongly reduce the magnetic attraction force, and continues by raising the heating power of the antagonist microactuators to decrease the bending angle towards negative values. If the power is switched off before a critical bending angle of about −80° is reached, the microactuator system adopts the metastable intermediate configuration (b’) at around 0°, as shown in Fig. 7b. Beyond −80°, magnetic latching by the permanent magnet underneath the demonstrator is used to stabilize the table configuration (c). The NiMnGa pads can self-latch when the heating power is reduced to allow for the recovery of the magnetic attraction force. This process can be reversed again by selective heating of the protagonist microactuators, which shows that the 3D shape of the demonstrator system can be reprogrammed without any manual intervention.

The microactuator system undergoes this sequence in both directions on demand by selective control of the SMA microactuators and local heaters. The course of shape reconfiguration occurs in a nonlinear and hysteretic manner, reflecting the complex kinetics of phase transformation of the SMA microactuators. Therefore, different intermediate paths are taken between the stable configurations depending on the heating signal. The return from table configuration (c) starts at a lower critical power compared to the pyramid configuration, as the distance between NiMnGa pads and external magnet is smaller in the table configuration, resulting in a smaller magnetic latching force. Fig. S7 in the Supplementary Materials also shows the time-resolved bending performance at different power levels, highlighting the power-dependent dynamics of folding actuation.

## Discussion

Reprogrammable matter is an important extension of programmable matter as it allows reconfiguring its 3D shape after initial folding without manual intervention. Reprogrammability becomes inevitable at miniature scales, as the manipulation of individual tiles like manual resetting is hardly possible. Here, we introduce novel concepts of bidirectional folding of tiles well below the millimeter range and of self-latching and -unlatching by combining smart materials and microtechnology, which enables the realization of reprogrammable micromatter. The presented origami-inspired reprogrammable microactuator system makes use of antagonistic SMA microactuators for bidirectional bending with an angular range of ±80°. Integrated soft-magnetic pads with low ferromagnetic transition temperature combined with local heaters allow to selectively switch magnetic latching forces. Due to the small edge length of the tiles of 500 µm, the different subsystems have to be integrated within a very limited space, which poses the following challenges:

1. The different subsystems have to be designed in parallel, taking into account thermal, mechanical, and magnetic coupling effects. At first, this includes the coupled design of the antagonistic SMA microactuators. Magnetron-sputtered TiNiCu films are used for actuation at a maximum strain and corresponding shape recovery stress of 2 % and 230 MPa, respectively. Thus, the strain is well above the minimal strain to induce detwinning, but still low enough to avoid plastic strain. The counteracting torque of the protagonist and antagonist microactuators in the unheated (martensitic) state should be as low as possible to enable large bending angles. Therefore, wing-like structures in the double-beam layout of the microactuators are designed to improve compliance and, thus, to increase the angular range of bending^30^. To ensure that the protagonist and antagonist microactuators can be controlled selectively, their temperature profiles caused by Joule heating and heat transfer are designed to be sufficiently homogenous, and their temperature difference is adjusted to be larger compared to the hysteresis of the phase transformation. Secondly, for the design of the magnetic latching system, TiNiCu meander structures are provided on each tile to form local heaters for indirect heating of soft-magnetic NiMnGa pads. In the current layout, the local heaters are electrically connected in series with the antagonist microactuators. The used magnetron-sputtered NiMnGa film materials show a low ferromagnetic transition temperature, and, thus, a small electrical power is sufficient for locally switching the magnetic attraction force between the NiMnGa pads and a NdFeB magnet. In the current design, magnetic latching/unlatching is demonstrated by using an external NdFeB magnet with an edge length of 400 µm. However, smaller permanent magnets with an edge length of 50 µm could be fabricated by magnetron sputtering^40^ and integrated directly on the tiles.
2. Another challenge is the development of a microfabrication process taking into account the specific requirements of processing the various materials and structures. We present a process flow involving three lithography steps. This includes the magnetron-sputtering and micromachining of TiNiCu films for microactuation and of NiMnGa films for switchable magnetic latching. The rigid tiles are fabricated monolithically by deposition and structuring of a 20 µm-thick SU-8 layer.
3. A dedicated procedure is required for the shape setting of each SMA microactuator. We present a procedure for local heat treatment of the SMA microactuators in constraint shape by Joule heating at temperatures above 400 °C to set their memory shape individually^41^. Thereby, the temperature-homogenized design of the microactuators helps to avoid large temperature gradients and to increase the reproducibility of the shape-set angle. Previous investigations report on the heat treatment of microscale wires and structured films by Joule heating using mechanical jigs^42,43^. A large temperature gradient may occur, which may affect the course of phase transformation^44^. Compared to homogeneous heating methods like furnace heating, the presented method offers a very local shape setting followed by rapid cooling due to natural heat convection and, thus, allows to functionalize the protagonist and antagonist microactuators for bidirectional bending. Further detailed studies will be needed to clarify the influence of the heat-treatment temperature and time on the functional fatigue^45^.

The presented microactuator system consists of a base tile and three movable tiles that can be programmed to adopt the 3D shape of a pyramid. Later on, the system can be reprogrammed to self-unlatch, -unfold, and adopt the shape of a table. The bidirectional folding performance is highly symmetric, which suggests that consistent shape-setting conditions have been realized for the protagonist and antagonist microactuators. The maximum folding angle reaches about ±100° due to magnetic latching. The torque produced during actuation ranges from 10¹ to 10² μNmm (Figs. 5e and f), which well exceeds the torque produced by gravity. By integrating the magnets with an edge length of 400 µm on the tiles, the torque generated by gravity would increase up to about 2 µNmm, which is still well below the torque produced during actuation. The presented folding range is significantly larger than that of previous bidirectional bending SMA actuators, while the actuator size is considerably smaller^29^. Previous bidirectional designs are mostly wire-based and macroscopic^46^. On the other hand, foil-based designs reach microscopic dimensions but are unidirectional up to now^47^. Thus, the presented reprogrammable microactuator system marks significant progress. Further extension of the bidirectional folding angle by SMA microactuators towards 360° requires more sophisticated designs and, thus, remains a challenge.

The effect of thermal cross-coupling plays a key role in the performance of the SMA microactuator system. We show that device angle and blocking torque will be reduced when exceeding the power range required for phase transformation. At large heating power, the torque decreases as thermal cross-coupling causes counter-actuation by the protagonist and antagonist microactuators. The allowable power range depends on the maximum folding angle and heating rate.

## Conclusions

We present an origami-inspired reprogrammable microactuator system consisting of antagonistic SMA microactuators for bidirectional folding of miniature-scale tiles and integrated heatable soft-magnetic pads for magnetic latching and unlatching. The base materials for microactuation are magnetron-sputtered TiNiCu films of 5 and 10 µm thickness that are operated at a maximum strain of 2 % and a corresponding shape recovery stress of 230 MPa. Magnetron-sputtered 5 µm-thick NiMnGa films used for switching magnetic latching forces have a low ferromagnetic transition temperature of 94 °C. A model-based design is presented using a thermodynamics-based SMA model and a linear regression algorithm to describe the spatial dependence of magnetic latching forces. Due to the close vicinity of the subsystems, a key design challenge is thermal cross-coupling. To ensure that the protagonist and antagonist microactuators can be controlled selectively, their temperature profiles caused by Joule heating and heat transfer are designed to be sufficiently homogenous, and their temperature difference is adjusted to be larger compared to the hysteresis of the phase transformation. Local heaters for switching of magnetic forces are designed for sufficiently low heating power below the power range required for bending actuation. A microfabrication process is developed, which includes a procedure for local shape setting of the SMA microactuators to adjust their memory shapes individually to functionalize their performance as protagonists or antagonists. The presented demonstrator system consists of four triangular tiles with an edge length of 500 µm. The microactuator system reversibly self-folds and -unfolds with an angular range of ±80°, which is extended to about ±100° by magnetic latching. The maximum torque of the microactuators is about 122 µNmm. We demonstrate that the microactuator system can be programmed to adopt the shape of a pyramid and subsequently reprogrammed to adopt the shape of a table without manual intervention.

The microactuator system can be further miniaturized, and the number of tiles can be increased using state-of-the-art microtechnology. An obvious extension to the basic performance of the presented reprogrammable microactuators system is the separate addressing of each tile to enable reprogramming the tiles individually. By integrating permanent micromagnets on the tiles and adding further movable tiles, the degrees of freedom of the system will increase considerably. This will enable a variety of functional shapes and motions based on reprogrammable folding. Possible applications are foreseen, e.g., in adaptive microoptics, microrobotics, and microfluidics. However, this approach involves additional challenges to be addressed in the future. Further miniaturization will increase the significance of thermal cross-coupling. Therefore, the use of SMA materials with narrow hysteresis width will become increasingly important to enable selective heating of the SMA microactuators. In addition, optimized protocols of heating power and time will be required to minimize heat dissipation and heat transfer. Upscaling the present design concept will lead to an increasing number of required interconnections, which will lead to constraints in size and number of tiles. Therefore, new concepts for the control of microactuators and local heaters have to be explored.

## Materials and methods

A thermodynamic model for the constitutive behavior of the SMA material has previously been developed to predict the time-resolved evolution of the coupled thermo-mechanical properties of SMA microactuators^48,49^. The model is based on the Helmholtz free energy with strain and temperature as control variables, and computes martensite volume fraction and transformation strain tensor as internal variables. In the hypo-elastic formulation of the strain rate additively composed into elastic and transformation strain rates, an updated Lagrangian description is applied that takes the large rotation in the bending of slender beams into account. The constitutive model is implemented in the FEM code ABAQUS in the form of a user-defined material subroutine. The FEM simulation considers a fixed central and a movable polymer tile being interconnected by a protagonist and an antagonist SMA microactuator as depicted in Fig. 3b–d. The geometries are meshed by Q3D20 20-node brick quadratic thermal-electrical-structural elements. A minimum of two elements across the SMA microactuator thickness is considered to ensure the accuracy of the contact algorithm and mesh. Table S1 in the Supplementary Material summarizes the thermal and electrical material parameters used in the FEM simulations.

## Supplementary information

Supplementary Material—Origami-Inspired Reprogrammable Microactuator System

## Data Availability

All data are available from the corresponding author upon reasonable request.
