# Supplementary material for: Origami-inspired reprogrammable microactuator system
Source: Microsyst Nanoeng. 2025 Oct 9;11:182. doi: 10.1038/s41378-025-01026-x (PMC12511558; doi:10.1038/s41378-025-01026-x)
Supplement: Supplementary file 1 — Supplementary Material—Origami-Inspired Reprogrammable Microactuator System [file 41378_2025_1026_MOESM1_ESM.pdf]

# Supplementary Material

## Origami-Inspired Reprogrammable Microactuator System

Vincent Gottwald[1], Lena Seigner[1], Makoto Ohtsuka[2], Rundong Jia[3], Pejman Shayanfard[3], Frank Wendler[3], Lars Bumke[4], Eckhard Quandt[4], and Manfred Kohl[1]

[1]Institute of Microstructure Technology, Karlsruhe Institute of Technology (KIT), Karlsruhe, Germany

[2]Institute of Multidisciplinary Research for Advanced Materials, Tohoku University, Sendai, Japan

[3]Institute of Materials Simulation, Friedrich-Alexander-Universität, Erlangen-Nürnberg, Germany

[4]Department of Materials Science, Kiel University, Kiel, Germany

Corresponding Author: Manfred Kohl[1], manfred.kohl@kit.edu , +49 721 608-22798

### A.1 Shape Setting Parameters

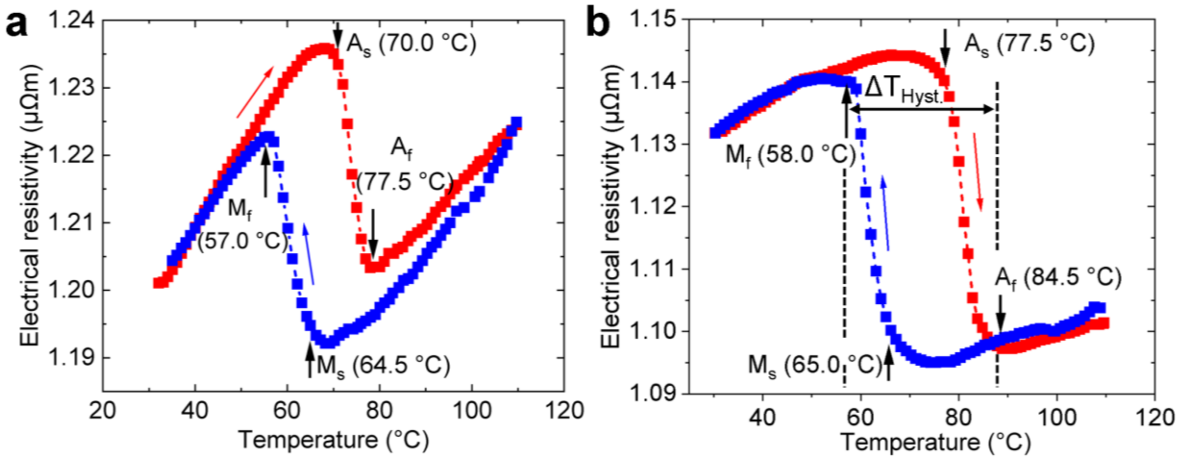


**Fig. S1:** Electrical resistivity versus temperature of TiNiCu test samples with dimensions of 10x5x0.01 $mm^3$ determined by the four-point measurement method. Quasi-stationary conditions are established by ramping the temperature stepwise and allowing for sufficient waiting periods between each step to ensure that the influence of temperature change is negligible. (a) Resistivity after fabrication by magnetron sputtering and rapid thermal annealing and (b) after additional local shape setting at 485 °C for 60 s. The start / finish temperatures of martensitic and reverse transformation $M_{s/f}$ and $A_{s/f}$ are determined using the tangential method.

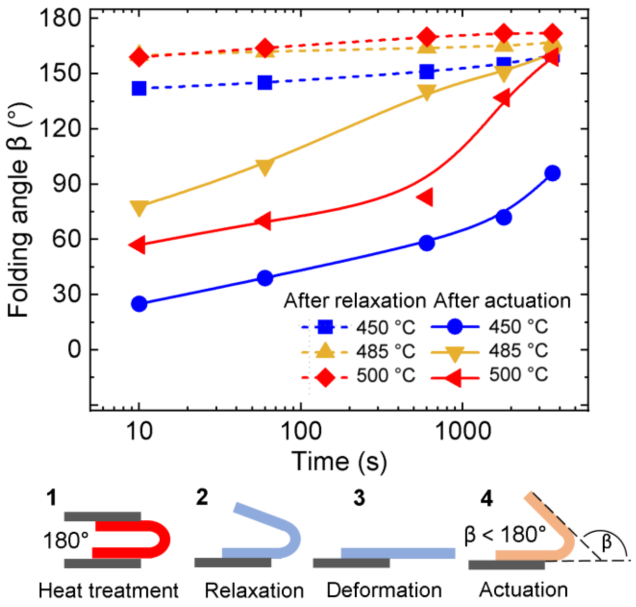


**Fig. S2:** Experimental study of maximum bending angles versus heat treatment time at different heat treatment temperatures of a unidirectional TiNiCu bending microactuator with a thickness of 10 µm. As illustrated in the inset, heat treatment is performed at a bending angle of 180° at a bending radius of 250 µm. Dashed lines represent the bending angles after release of the mechanical constraint of shape setting and relaxation during cooling back down to room temperature (2). Solid lines represent the bending angles after resetting the bending angle to 0° (3) and subsequent actuation by Joule heating above $A_f$ temperature (4). In the study of the microactuator system, we select a heat treatment time and temperature of 60 s and 485 °C, respectively. The shorter heat treatment time is sufficient to achieve an angular range of bidirectional bending actuation of ±80°. Thereby, degradation effects due to oxidation and diffusion are minimized.

### A.2 Thermal Cross-Coupling

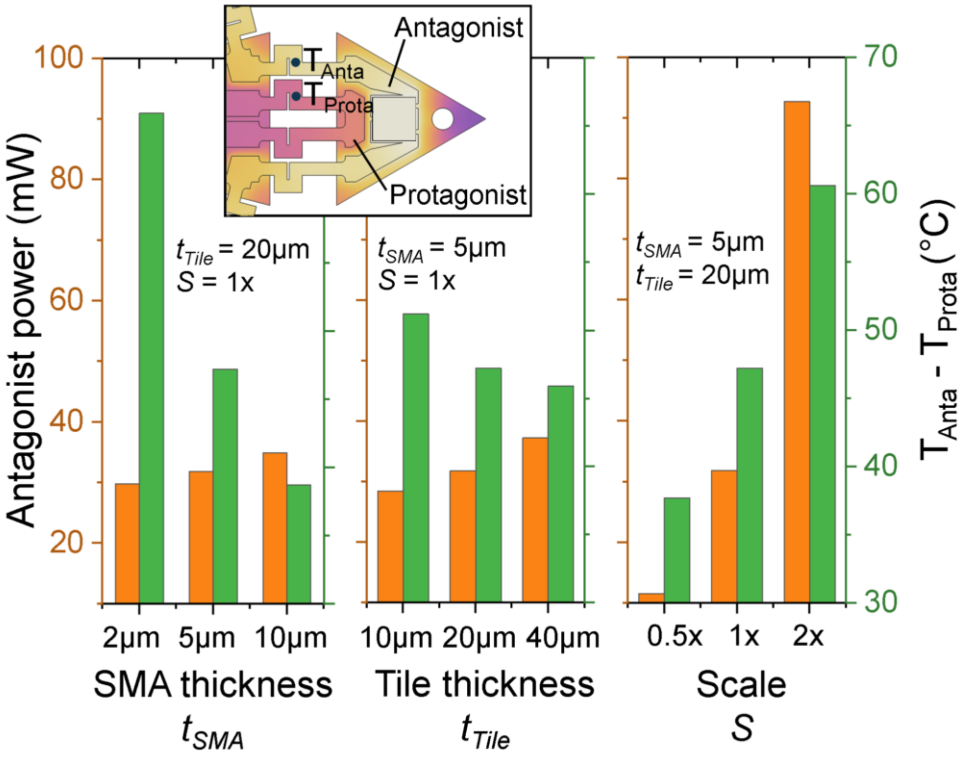


**Fig. S3:** FEM simulation study of thermal cross-coupling due to heat conduction and convection by comparing the temperatures in measurement points $T_{Prota}$ and $T_{Anta}$ of the protagonist and antagonist microactuator, respectively (see inset), after Joule heating the antagonist actuator above the phase transformation temperature $A_f$ with the power indicated in orange. The temperature difference $T_{Anta}$ -$T_{Prota}$ indicated in green is presented for three different SMA thicknesses $t_{SMA}$, SU-8 tile thicknesses $t_{Tile}$, and the overall dimensions of the SMA microactuator given by the scaling factor $S$. A temperature difference larger than 30 °C is required to thermally decouple the protagonist and antagonist microactuator sufficiently.

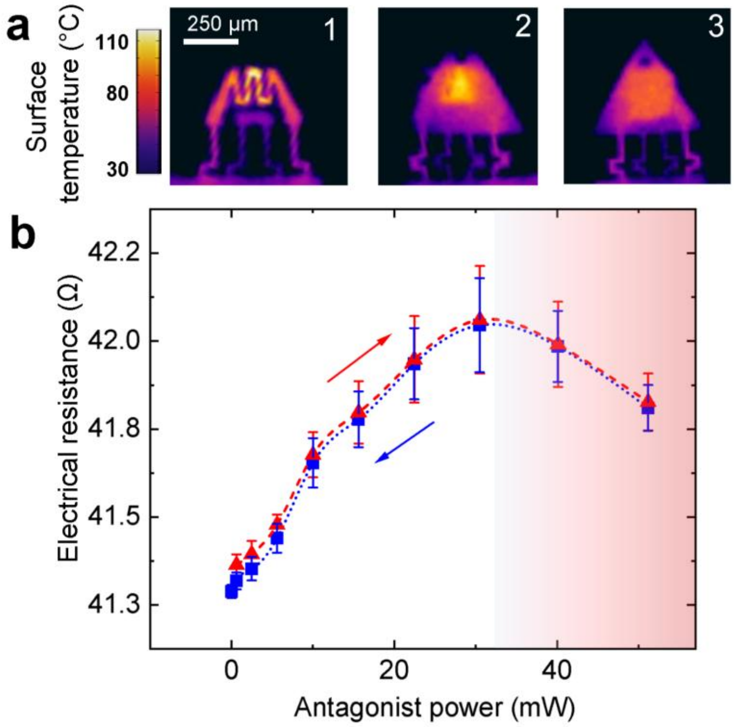


**Fig. S4:** Experimental study of thermal cross-coupling of the SMA microactuator system by measuring the electrical resistance of the protagonist microactuators while heating the antagonist microactuators. A decline of electrical resistance indicates the onset of martensite-to-austenite phase transformation and, therefore, the critical power for thermal cross-coupling. (a) IR images determined for a single tile in air at a heating power of 30 mW: temperature profile of antagonist microactuator and local heater in the absence of polymer tile (1), including polymer tile (2) and including polymer tile and NiMnGa pad (3). (b) selective heating of antagonist microactuators (Red - heating, blue – cooling). The critical power is about 32 mW. This highlights the importance of precise power control to limit simultaneous actuation of protagonist and antagonist.

**A.3 Modeling of Magnetic Latching Force**

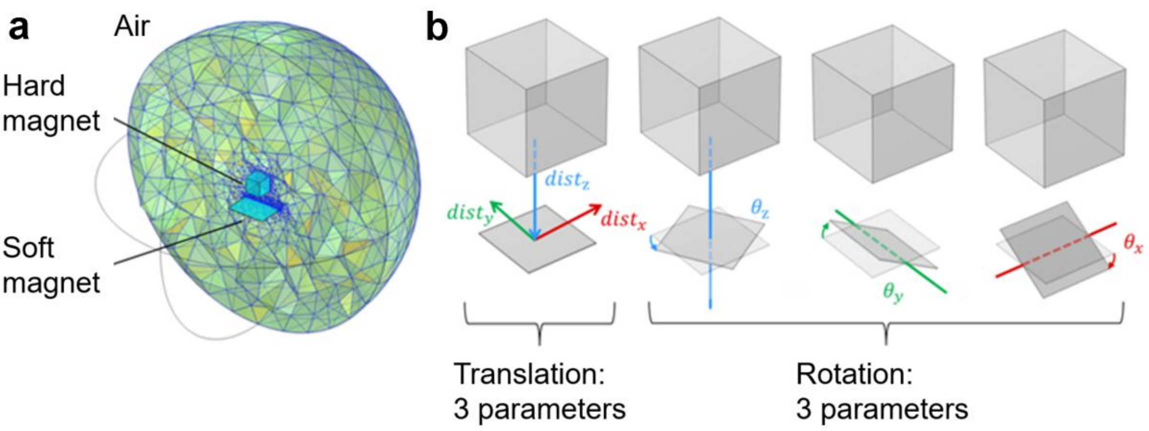


**Fig. S5**: To incorporate the magnetic attraction force in the system simulations, a function depending on distance and relative position between the bulk magnet and NiMnGa pad is determined by a regression technique. (a) illustrates the simulation setup (COMSOL multiphysics), which includes three domains: a cubic hard magnet (400 × 400 × 400 µm³), a soft magnet (350 × 350 × 5 µm³), and a sufficiently large spherical air domain to accommodate the magnetic field. Other materials are left away due to their negligible inductivity. The input data for regression comprises the simulated force vector from a large number of magnetostatic simulations combining a raster of the three translational and three rotational degrees of freedom (DOFs), as depicted in (b): translation along x, y, z axes and rotation angles around x, y, z axes (centered on the soft magnet). In detail, the raw dataset comprises $n$ samples, with $n$ representing the number of variable sets ($n = 52{,}889$ for this regression). Each set consists of 3 translational and 3 rotational features along and around the x, y, and z axes as input data and the 3 latching force components in x, y, and z directions as outputs. The objective is to construct an explicit polynomial function $\mathrm{f}^{(\mathrm{k})}(\mathrm{x})$ for each output $y^{(k)}$ ($k = 1, 2, 3$), capturing the relationship between input features and target outputs. To address potential nonlinear interactions among input features, a polynomial basis expansion method[1,2] is employed to map the original 6-dimensional feature space into a higher-dimensional polynomial space for upcoming linear regression.

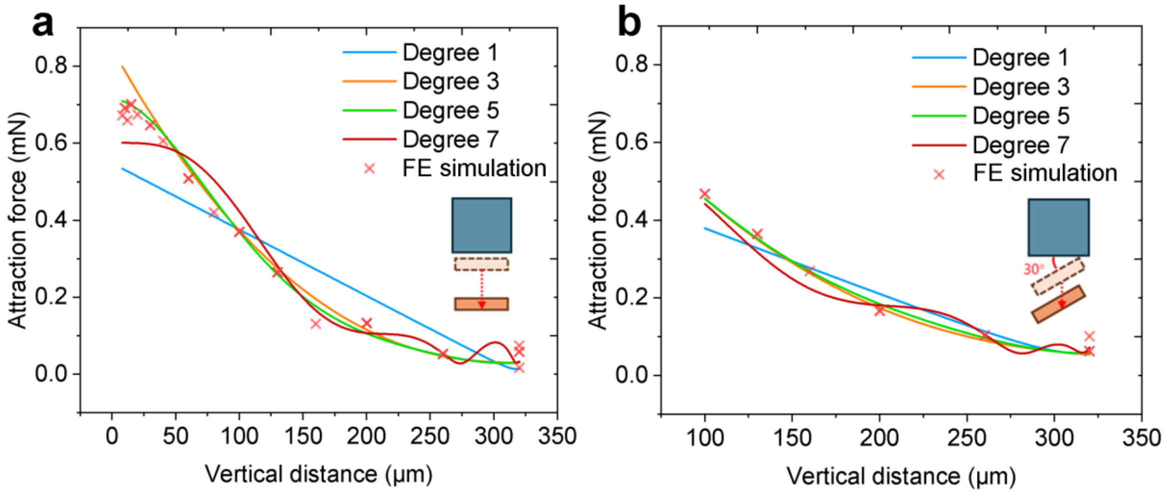


**Fig. S6:** Finite element (FE) simulations (COMSOL) and regression model predictions of magnetic latching forces versus vertical separation distances between the soft and hard magnets. (a) Hard and soft magnets are parallel and center-aligned and (b) rotated by 30° around the x-axis. The scatter points denote the FE simulated force magnitudes, whereas the solid lines represent the predictions of the regression model for different polynomial degrees. Linear models (degree 1) exhibit limited capability to capture the inherent nonlinearity of the force-distance relationship. By incorporating polynomial-expanded features (degrees 3 and 5), the models effectively capture the nonlinear variations in latching force as the vertical distance increases. While higher polynomial degrees enhance the model's ability to fit complex patterns, they also increase sensitivity to outliers. Notably, excessively high degrees (degree 7) induce overfitting, showing an unrealistic oscillatory behavior (wave-like fluctuations) in predictions. Overall, the model can closely follow the underlying trends of the simulated forces while avoiding spurious oscillations, demonstrating robustness in predicting nonlinear magnetic interactions.

### A.4 Actuator Dynamics

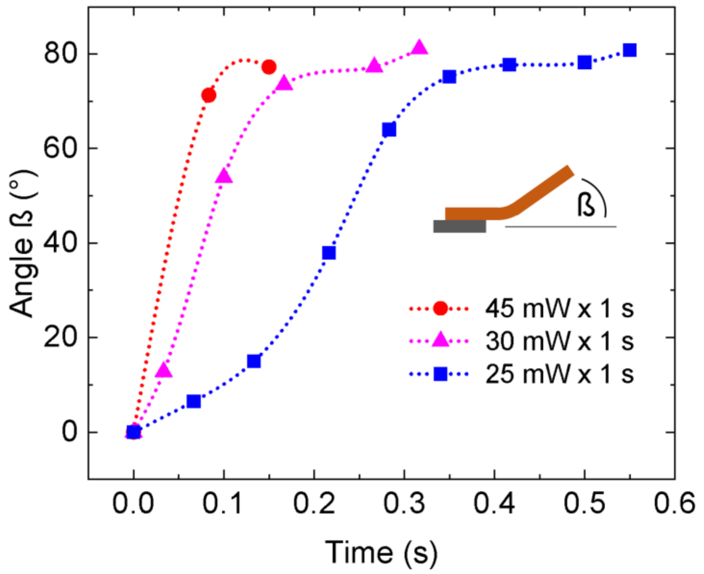


**Fig. S7**: Angle versus time at different power levels of a single protagonist microactuator being part of the reprogrammable origami-inspired microactuator system. The heating power is applied as a step-function with time duration of 1s while the dynamic response is recorded with a microscope camera and evaluated with ‘Tracker” analysis software. The response time to reach 80° decreases with increasing power. At 45 mW, the maximum angle is reached within about 100 ms.

### A.4 Material Parameters

**Table S1:** Thermal and electrical material parameters used in the FEM simulations.

| **Material Parameter** | **Value** | **Reference** |
|---|---|---|
| SU-8 3000 - Material density | 1200 kg $m^{-3}$ | [3] |
| SU-8 3000 - Thermal conductivity | 0.2 W $m^{-1}$ $K^{-1}$ | [3] |
| SU8-3000 - Heat capacity | 1500 J $kg^{-1}$ $K^{-1}$ | [3] |
| SU-8 3000 - Electrical resistivity | 7.8 x $10^{14}$ Ω cm | [4] |
| TiNiCu - Martensite start / finish temperatures $M_s$ / $M_f$ | 327 / 322 K | This work |
| TiNiCu - Austenite start / finish temperatures $A_s$ / $A_f$ | 337 / 342 K | This work |
| TiNiCu - Heat capacity | 360 J $kg^{-1}$ $K^{-1}$ | This work |
| TiNiCu - Material density | 6225 kg $m^{-3}$ | [5] |
| TiNiCu - Electrical resistivity in austenite / martensite state | 1.14 x $10^{-6}$ / 1.1 x $10^{-6}$ Ωm | This work |
| Elastic module in austenite state | 45 GPa | This work |
| Elastic module in martensite state | 20 GPa | This work |
| Critical stress at the start of martensite reorientation | 75 MPa | This work |
| Critical stress at the end of martensite reorientation | 120 MPa | This work |
| Stress rate of martensite transformation temperature (Clausius-Clapeyron coeff.) | 14.5 MPa $K^{-1}$ | This work |
| Stress rate of reverse transformation temperature (Clausius-Clapeyron coeff.) | 17.0 MPa $K^{-1}$ | This work |
| Maximum transformation strain | 0.015 | This work |

**References**


1. Hastie, T., Tibshirani, R. & Friedman, J. *The Elements of Statistical Learning*. (Springer New York, New York, NY, 2009). doi:10.1007/978-0-387-84858-7.
2. Bishop, C. M. *Pattern Recognition and Machine Learning*. (Springer, New York, 2006).
3. El-Ali, J. *et al.* Simulation and experimental validation of a SU-8 based PCR thermocycler chip with integrated heaters and temperature sensor. *Sensors and Actuators A: Physical* **110**, 3–10 (2004).
4. Melai, J., Salm, C., Smits, S., Visschers, J. & Schmitz, J. The electrical conduction and dielectric strength of SU-8. *Journal of Micromechanics and Microengineering* **19**, 065012 (2009).
5. Cirstea, C. D. *et al.* Studies about structural and thermal investigations on Ti50Ni30Cu20 alloys obtained by different technological processes. *Rom. J. Phys* **66**, 601 (2021).
